# Modelling maximum cyber incident losses of German organisations: an empirical study and modified extreme value distribution approach

**DOI:** 10.1057/s41288-023-00293-x

**Published:** 2023-04-13

**Authors:** Bennet von Skarczinski, Mathias Raschke, Frank Teuteberg

**Affiliations:** 1 PricewaterhouseCoopers, Fuhrberger Strasse 5, 30625 Hannover, Germany; 2 Ecclesia Re, Cologne, Germany; 3 University Osnabrueck, Katharinenstrasse 3, 49074 Osnabrueck, Germany

**Keywords:** Cyber incident losses, Loss size distribution, Extreme value distribution, Tapered distribution, Information security management, Data breach

## Abstract

Cyber incidents are among the most critical business risks for organisations and can lead to large financial losses. However, previous research on loss modelling is based on unassured data sources because the representativeness and completeness of op-risk databases cannot be assured. Moreover, there is a lack of modelling approaches that focus on the tail behaviour and adequately account for extreme losses. In this paper, we introduce a novel ‘tempered’ generalised extreme value (GEV) approach. Based on a stratified random sample of 5000 interviewed German organisations, we model different loss distributions and compare them to our empirical data using graphical analysis and goodness-of-fit tests. We differentiate various subsamples (industry, size, attack type, loss type) and find our modified GEV outperforms other distributions, such as the lognormal and Weibull distributions. Finally, we calculate losses for the German economy, present application examples, derive implications as well as discuss the comparison of loss estimates in the literature.

## Introduction

Information security (IS) risks and incidents have risen in recent years, with considerable economic and social consequences for public and private organisations as well as individuals and society at large (OECD 2015; DCMS 2020; Buil-Gil et al. 2021). The need to quantify, model and manage IS risks, also referred to as cyber risk, is thus becoming increasingly important.

The 2022 Allianz Risk Barometer considers IS incidents as the most crucial business risk for organisations worldwide (Allianz 2022). In the U.K. the proportion of organisations whose senior management considers IS a high-priority topic and subsequently increased IS risk identification and management activities has risen to 80% (DCMS 2020). In another recent global survey, nearly 60% of participants, mainly business, technology and security executives of larger organisations, are starting to financially quantify cyber risks to improve ISM capabilities, while another 17% are planning to do so in the next two years (PwC 2021).

Despite the extensive nature of the topic, there is a huge lack of reliable empirical data (Marotta et al. 2017; Wolff and Lehr 2017; EIOPA 2018; Romanosky et al. 2019; Dambra et al. 2020; Eling 2020; Wrede et al. 2020; Buil-Gil et al. 2021; Cremer et al. 2022; Wheatley et al. 2021), which prevents organisations from better calculating IS investments and deciding on cyber risk transfers as well as enabling insurance companies to more precisely price their cyber policies.

The reasons for this lack of empirical data are manifold. Besides the fact that police statistics do not yet capture the phenomenon in a consistent manner, and organisations are hesitant to disclose their own cyber challenges, there is one striking problem: cyber risk is not trivial, it is difficult to measure and it is extreme in many ways.

On the one hand, the media regularly report on cyberattacks with massive impact, such as the ransomware attack that shut down 800 Swedish grocery stores (Ahlander and Menn 2021) or the loss of 40 terabytes of confidential data of the German Automotive Tier 1 supplier Continental (Julianto 2022). On the other hand, the rare empirical studies that are available report that, for the majority of organisations, comparatively little harm results from cyberattacks (Rantala 2008; Richards 2009; Paoli et al. 2018; DCMS 2017, 2019, 2020, 2021, 2022).

As well as the small number of studies collecting cyber loss data, there have also been a few studies modelling cyber incident losses based on empirical data. Many of them have found that negative outcomes and losses from cyber incidents are highly skewed and that heavy tails follow power law distributions (Edwards et al. 2016; Kuypers et al. 2016; Riek et al. 2016; Wheatley et al. 2016, 2021; Eling and Wirfs 2019; Strupczewski 2019; Jung 2021).

As an example, Wheatley et al. (2016) found that the size of public personal data breaches from 2000 to 2015 can be well modelled by a truncated Pareto distribution. Strupczewski (2019) suggests applying extreme value theory and a generalised Pareto distribution (GPD) to model extreme cyber losses. Jung (2021) finds stationarity, the presence of autoregressive features and the Frechet type of generalised extreme value distribution (GEV) to be the most appropriate solution to model data breach loss maxima series.

However, all previous findings on empirical modelling of cyber losses are built on unassured data sources. Edwards et al. (2016), Kuypers et al. (2016), Riek et al. (2016), Wheatley et al. (2016), Eling and Wirfs (2019), Strupczewski (2019) and Jung (2021) use public op-risk databases for which the consistency, quality, representativeness and completeness of the data for the underlying population cannot be ensured. This weakness of op-risk databases, which are especially incomplete for small and medium-sized enterprises (SMEs) and smaller events, can be addressed by representative surveys (Wheatley et al. 2021).

Against this background, our article contributes two aspects to the body of knowledge. First, to our knowledge, it is the first paper to model cyber losses based on a representative, large-scale, computer-assisted telephone survey using a stratified random sample that differentiates several organisational characteristics for one of the leading industrial economies worldwide. Although telephone interviews also have limitations, which we discuss in the “Discussion” section (e.g. self-reporting, social desirability), the knowledge of the population as well as transparency and control over the random sample drawing reduce the over-representativeness of large cyber incidents as is common in op-risk databases. In particular, our focus on SME covers the blind spot of op-risk databases and validates the findings of previous research. Second, we propose a modified GEV distribution approach, to appropriately describe the tail behaviour of cyber incident losses. Instead of exploring the internal causalities of cyber losses, in this article we focus on describing and modelling loss distributions according to certain characteristics based on a well-controlled sample.

According to our dataset, which explicitly asks for details on the most serious cyber incident in the last 12 months, we use extreme value theory (EVT), which naturally focusses extreme manifestations. Besides the irrefutable reason that our entire sample consists of extreme values, previous research has also argued to apply EVT to model cyber losses (Strupczewski 2019). Besides our focus on the GEV, we also considered other distributions in our preparation phase, such as the normal distribution, gamma or GPD distribution, but sorted them out from the beginning due to a very weak fit (graphical analysis: PP plots and survival functions). We discuss the issue of interdependence between cyber losses but argue it plays a subsequent role for our analysis because many organisations now have a basic level of security against mass attacks (DCMS 2019) and, at the same time, targeted attacks in particular lead to extreme losses, which usually aim at single organisations. Furthermore, there are data-historical and statistical reasons, which we discuss in the “Data analysis approach, distribution models and inference” section.

We derive three research questions (RQs). For such extremely distributed damage events as cyber incidents, it is important to model the tail as best as possible, since the tail behaviour strongly influences the risk of extreme cyber incident losses according to EVT and statistics (Coles 2001; Beirlant et al. 2004). Thus, according to the 80/20 rule, IS risk management approaches should focus on tail behaviour (Strupczewski 2019). According to more recent research in actuarial science, alternatives to the Pareto distribution or GPD are in focus to model the distribution of insured loss and claim sizes (Schoenberg and Patel 2012; Albrecher et al. 2021; Raschke 2020). Such alternatives are called ‘tapered’ or ‘tempered’ Pareto distributions and, to our knowledge, have not yet been applied to GEVs in general, and cybersecurity management in particular. Therefore, we hypothesise: *How could a GEV be modified in order to better model the tail of cyber incident losses?* (RQ1). In view of the absence of reliable samples underlying previous modelling attempts, we used a basis of a stratified random sample of 5,000 German organisations for analysis. *What distribution types best model the losses of the most severe cyber incident in the last 12 months, and what differences exist with respect to company size, industry, loss type or attack type?* (RQ2). Based on our modified GEV loss distributions, we analyse: *What losses can be derived for an average organisation and the whole German economy?* (RQ3).

We structure our article as follows. In the “Survey methodology and sample” section, we describe our survey and sample. The “Operationalisation of cyber incident losses” section includes our operationalisation of cyber incident losses. In the “Data analysis approach, distribution models and inference” section, we discuss the issue of interdependence, deduce how we analyse our data and, on a conceptual level, respond to RQ1. Our empirical results addressing RQ2 and RQ3 are provided in the “Empirical results” section, followed by a discussion on what implications can be drawn for practice and academia as well as the limitations of our paper. Finally, the “Conclusions” section summarises our findings.

## Survey methodology and sample

In this section, we describe the sampling and survey approach and illustrate the data quality measures taken. In the context of this article, we are reusing data stemming from the government-funded research project ‘Cyberattacks against companies’ (Dreissigacker et al. 2020), which carried out computer-assisted telephone interviews (CATI) with representatives of 5,000 German organisations with more than nine employees between August 2018 and January 2019.

### Sample

Organisations with more than nine employees that operate as independent legal entities and are located in Germany form the population (*n* = 372,599). Since micro-organisations are subject to comparatively strong changes (e.g. insolvency, registration), which has a negative impact on the availability of contact information in the sampling databases used, they were excluded. Two commercial company databases (Bisnode; Heins & Partner) were used for sampling. They provided contact information and contact persons, as well as the sector affiliation according to the German WZ08 classification, which allows for international comparison with other official statistics.

In 2017, around 3.5 million organisations were registered in Germany, of which 89% were organisations with less than 10 employees. Focusing on the remaining 11% (*n* = 372,599) organisations), the greatest part are organisations with 10–49 employees (79%), whereas organisations with more than 250 employees only make up 4% of the organisations in Germany. Nevertheless, the organisations in our sample employ around 82% of all employees in Germany (Dreissigacker et al. 2020).

Interviewees were mainly single individuals in charge of IS and/or IT (67%), management board members (24%) and other representatives of the organisation (e.g. data protection, plant security, audit; 9%). A disproportionately stratified random sample was used (Table 1) to ensure that subpopulations of interest (e.g. large organisations) are appropriately represented in the sample. Even though we are able to reproportionalise our sample using employee class and sector weights, we did not do this in our models because sector and employee class are separately controlled for. We contacted 43,219 organisations to reach our sample of 5,000 participants (participation rate: 11.6%) and have no indications of structural distortions with regard to company characteristics in the population and sample. Further information on the sample used can be found in Dreissigacker et al. (2020).

**Table 1:** Sample by employee class

Employees	Amount (N)	Portion (%)
10–49	1190	24
50–99	1181	24
100–249	1120	22
250–499	1005	20
> 500	504	10
Total	5000	100

### Questionnaire and CATI-method

The questionnaire was derived from expert interviews with two insurance companies and six practitioners from German cybersecurity-related authorities (Stiller et al. 2020). Moreover, a broad literature review and input from a regional business advisory council shaped the questionnaire (Dreissigacker et al. 2020). In comparison with web or postal surveys, the favoured target interviewees (e.g. IT or executive management) could be reached more concisely. The support from experienced and instructed interviewers helped to answer inquiries immediately, which has a positive effect on the general data quality (Steeh and Charlotte 2008). Additionally, by means of computer support and sophisticated filter guidance, the inquiry could be performed efficiently (Lavrakas 2008). Moreover, telephone interviews using list samples demonstrated reasonable participation rates (Steeh and Charlotte 2008). Furthermore, representative surveys address the weakness of op-risk databases, being incomplete especially for SME-related and smaller cyber incidents (Wheatley et al. 2021). Representative crime victimisation surveys have long supplemented the situation of incomplete official or police crime statistics (Mayhew and Hough 1992; Dijk and Mayhew 1992).

The questionnaire can be found in Dreissigacker et al. (2020). It contained 40 questions on interviewees’ risk perceptions, cyberattacks detected within the last 12 months, technical and organisational security measures deployed as well as demographic characteristics of the organisation. In addition, there was a focus on more detailed questions about the most severe attack in the last 12 months and its consequences.

### Survey conduction

The CAT interviews were conducted by a professional and certified survey institute, which was selected through an official Europe-wide tender offering. Pretests prior to the survey were carried out in two steps: first, by discussions with the project council, and second, by interviews with six further IT employees from organisations of various size and from various industries. Interview training sessions in two on-site call centres were conducted with the 141 interviewers prior to the field phase. Brief and clear questions were deliberately formulated to enable simple comprehension. The questionnaire was designed to last no more than 20 minutes to avoid fatigue effects. With the goal to boost participation among the contacted organisations, an official motivation letter of the German Federal Ministry for Economic Affairs and Energy was offered during the preparation phase. Furthermore, the questionnaire, if desired, was provided to the participants prior to the interview. Solely scientific use of the data and complete anonymity were guaranteed. In order to address ethical standards in IS research, the principles of the Menlo Report were respected (DHS 2012).

## Operationalisation of cyber incident losses

**Fig. 1:**
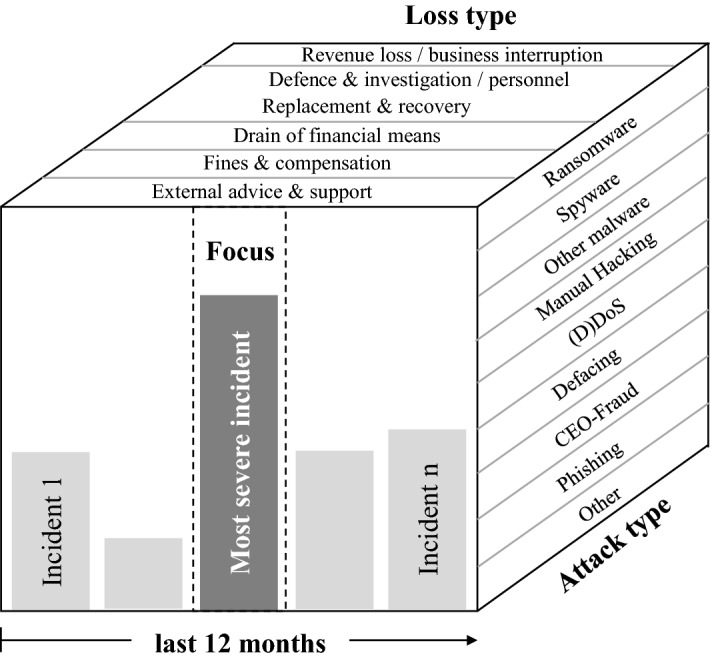
Operationalisation of losses

In the following, we describe our operationalisation of cyber incidents and associated losses. Since we are reusing the dataset of an existing research project, the following operationalisation is based on and reused from previous publications (Dreissigacker et al. 2020; von Skarczinski et al. 2021; von Skarczinski et al. 2022a, b). Defining IS, we presume that an external or internal threat initiates a cyberattack that is either blocked by a security control/measure or leads to an IS/cyber incident by exploiting a vulnerability, which thus causes consequences for an organisation. We define cyber incidents, initiated by cyberattacks, as intentional attacks against organisations that destroy, disable, disrupt or maliciously control a computing environment/infrastructure, destroy the integrity of the data, or steal controlled information (NIST 2020). The objectives of IS, availability, confidentiality and integrity of data, systems and processes are thus no longer ensured (ENISA 2017). Thus, we focus on actual cyberattacks and leave out technical malfunctions or procedural flaws that could also cause cyber losses. Our loss data can be distinguished with respect to three dimensions (Fig. 1).

### Attack type

Our dataset distinguishes nine attack types (Fig. 1), which were primarily derived from the established Commercial Victimization Survey of the UK Home Office (U.K. Home Office 2018). Besides ransomware and spyware, other malware was grouped together (i.e. scareware, viruses, worms, etc.) Manual hacking includes hardware manipulation and unauthorised configuration. Moreover, (distributed) denial of service attacks, defacing of web content, phishing as well as CEO fraud including similar social engineering approaches was differentiated.

### Loss type

We differentiate six loss types that can be assigned to either losses as a consequence of cyberattacks or losses that occur when responding to attacks. Excluding IS operating costs as well as indirect costs (e.g. reputational damage), our analysis focuses on losses directly assignable to a specific cyber incident, either cash effective or opportunity costs. The loss types were derived from the Commercial Victimization Survey of the UK Home Office (U.K. Home Office 2018). External advice and support includes costs for external emergency, forensics or legal support services, while fines and compensations include fees paid to customers, business partners or authorities. Replacement and recovery costs relate to software as well as hardware. Defence and investigation costs address internal action and personnel costs. We have not included an ‘other’ category for losses, as we assume that the listed loss types are exhaustive categories and that any other types are outside the scope of our analysis. Even though reputation risks might follow cyber incidents, we excluded them from our analysis because they are hard to identify and measure (Bandyopadhyay et al. 2009; Gatzert et al. 2016; Franke 2017).

All loss types were estimated in EUR by the interviewees. Reported total losses refer to all six loss types, while only observations that gave valid responses (no losses or losses > EUR 0) to all six loss types were included. Those ‘secured losses’ prevent an underestimation of total losses in case an organisation reported only some loss types, while suffering but not reporting other loss types.

### Most severe incident

Our analysis is focused on losses of a single cyber incident within the last 12 months that participants, based on their professional judgement, perceived as most severe considering the loss types outlined previously. This, in contrast to the last incident, enables us to model maximum losses. In terms of data quality, we assume interviewees will best remember the most severe incident as well as give more accurate information than they would offer if having to account for a certain time period including diverse incidents and attack types.

## Data analysis approach, distribution models and inference

In this section, we discuss the issue of interdependence and conceptualise the distributions to be modelled, including our modified extreme value distribution, as well as deduce how we analyse our data.

### Interdependence of cyber losses

In the context of cyber risk analysis, the issue of interdependence between cyber events can play an important role, since dependence could affect the statistical inference. We argue that interdependence plays a subsequent role for our data and analysis for the following reasons.

First of all, many organisations now have a basic protection level against mass attacks (DCMS 2019), which prevent organisations of an entire economy from being affected by non-specified attack vectors. Pertinent research suggests that the amount of harm from a cyber incident is not only dependent on external factors, but is strongly determined by an organisation's behaviour, structural characteristics and security measures (Hall et al. 2011; Liu et al. 2015; Biswas et al. 2016; Edwards et al. 2019; McLeod and Dolezel 2018; von Skarczinski et al. 2022a, b). If one looks at the last major cyber incidents in the media, targeted attacks aiming at single organisations or services in particular led to extreme losses (e.g. Continental (Glover 2022), Coop (Abrams 2021), Colonial Pipeline (Tidy 2021)). We are not aware of any attack wave that has ever affected an entire economy. According to Dreißigacker et al. (2021), in 2019/2020, the share of respondents that stated the extremely dangerous Emotet malware as the most serious attack in the last 12 months was 11.6%, with 98.4% of respondents stating that they suffered no or very little damage from the most serious attack.

Second, from a statistical perspective, the following aspects support our assumption of a lower importance of interdependence. A simple scaling of sample means to the sum of extreme losses of the entire finite population is not affected by a correlation between these variables since the expectation of the sum of random variables is the sum of their expectations (in both cases, with or without correlation). The point estimation of an expectation is not biased by an (auto)correlation, only the corresponding standard error becomes higher (e.g. Zwiers & Storch 1995). The parameter estimation of extreme value distribution is also less affected by (auto)correlation if the sample size is high (Landwehr et al. 1979).

In order to ensure our analysis is not negatively affected, we estimate the potential impact of interdependence between loss events in organisations (Annex D). Therein, we simulate the extremes of associated Poisson point processes. This approach is based on Schlather’s (2002) second theorem for max-stable random fields. It is related to spectral representation of extremes (Haan 1984) and the corresponding pseudo-polar coordinates (Coles 2001). Raschke (2022) realised further potential for the modelling of return periods of catastrophes, also focusing on the return periods that can be easily transformed to other (marginal) point processes (similar to the well-known copula approach or random variables). The working party of the German actuarial union uses the approach in a suggestion for the modelling of cumulus from associated cyber losses (Frey et al. 2022).

According to our modelling and conservative simulation in Annex D, the influence of interdependence to the distribution of annual loss maxima per organisation is small. The influence on the maxima of the entire population is higher, but our aim is to only estimate the dimension of this extreme loss. To our knowledge, this is the first simulation of its kind, since the issue of interdependency has not been considered further in previous related literature.

### Statistical background

An issue of modelling single maximum cyber incident losses is the probability mass for non-measurable random losses, $X$, during an attack. This can be considered in two ways. At first, the cumulative distribution function (CDF) $G(x)$ is used, which describes all losses, including negative ones. Since negative cyber incident losses do not exist in the real world, the issue is solved by using the value $G({x}_{T})$ for threshold ${x}_{T}$ to represent the probability mass, $P$, for all non-losses $X\le {x}_{T}$ with

1 $$P\left(X\le {x}_{T}\right)=G\left({x}_{T}\right).$$

Thus, only losses $X>{x}_{T}$ are observed in our analysis. Our threshold is ${x}_{T}=0$. The ‘unobserved’ losses are processed as censored observations. In this instance, the logarithm of the likelihood function for $n$ observations with indicator function ${\mathbb{I}}$ and probability density function (PDF) $g$, a first derivate of $G$, with parameter vector $\theta$ is:

2 $$Log\left(L\left(\theta \right)\right)={\sum }_{i=1}^{n}{\mathbb{I}}\left({x}_{i}\le {x}_{T}\right)log\left(G\left({x}_{T},\theta \right)\right) +{\mathbb{I}}\left({x}_{i}>{x}_{T}\right)log\left(g\left(x,\theta \right)\right).$$

As a second way to consider the issue, the probability mass is parametrised separately by

3 $${P}_{T}=P\left(X\le {x}_{T}\right).$$

Subsequently, all actual observed losses with $X>{x}_{T}$ are described by the conditional CDF $F(x|x>{x}_{T})$. The unconditional distribution is:

4 $$G\left(x\right)={P}_{T}+F\left(x|x>{x}_{T}\right)\left(1-{P}_{T}\right), x\ge {x}_{T}.$$

The corresponding logarithm of the likelihood function is:

5 $$Log(L(\theta ,{P}_{T}))={\sum }_{i=1}^{n}{\mathbb{I}}\left({x}_{i}\le {x}_{T}\right) log\left({P}_{T}\right) +{\mathbb{I}}\left({x}_{i}>{x}_{T}\right) log\left((1-{P}_{T})f\left(x|x>{x}_{T},\theta \right)\right).$$

It should be noted that any CDF $G(x)$ for a maxima implies a function for the expected exceedance frequency $\Lambda$ of underlying point events $X>x$ when we assume a Poisson point process:

6 $${\Lambda }\left( x \right) = - {\text{log}}\left( {G\left( x \right)} \right).$$

This means the non-exceedance probability for threshold *x* of the maximum equals the probability that not one point event *X* is larger than *x*. The latter approximately follows a Poisson distribution. In detail, the positive random integer $K$ is the number of events $X>x$ during a model period (one year) and has the Poisson distribution (Poisson 1837) (with intensity $\Lambda$ from Eq. (6))

7 $$P\left(k\right)=\frac{{\Lambda }^{k}\text{exp}(-\Lambda )}{k!}.$$

The relation (6) is mentioned by Coles (2001, Sect.  3.3.5, parameter ‘*y*_*p*_’) and it is more popularly known as ‘Poisson approximation’ in extreme value statistics (Coles 2002; Beirlant et al. 2004).

### Considered distribution models

Since the survey participants were interviewed about losses of the most severe cyber incident in the last 12 months, we analyse loss maxima. During our pre-analysis phase, we have considered several distribution models, such as the normal distribution, generalised Pareto distribution and gamma distribution, but according to graphical analysis (PP plots and survival functions) they were far from the distributions we introduce in the following.

The ‘natural’ distribution model for $G(x)$ is the established generalised extreme value (GEV) distribution (Beirlant et al. 2004) with the extreme value index $\gamma$, scale parameter $\sigma$ and location parameter $\mu$ for the CDF:

8 $$G\left(x\right)=\begin{array}{ll}exp\left(-{\left(1+\gamma \frac{x-\mu }{\sigma }\right)}^{-1/\gamma }\right), &\quad if \gamma \ne 0\\ exp\left(-exp\left(-\frac{x-\mu }{\sigma }\right)\right), & \quad if \gamma =0.\end{array}$$

The special case in which $\gamma =0$ is the well-known Gumbel distribution (Gumbel 1935). This distribution is max-stable, meaning that the maxima of $n$ independent realisations of $G\left(x\right)$ have the CDF ${G\left(x\right)}^{n}$. This is the same distribution as (8) after a linear transformation of either of the maxima or the parameters in (8). For the latter, the following applies:

9 $${\gamma }_{n}=\gamma ,{\sigma }_{n}=\sigma {n}^{\gamma },{\mu }_{n}=\mu -\frac{\sigma }{\gamma }\left(1-{n}^{\gamma }\right).$$

The survival function of the GPD is inside the outer exponential function in (8) and defined as (Beirlant et al. 2004):

10 $$\overline{H}\left( x \right) = 1 - H\left( x \right) = \begin{array}{ll} {\left( {1 + \gamma \frac{x - \mu }{\sigma }} \right)^{{ - \frac{1}{\gamma }}} , if \gamma \ne 0 } \\ {\exp \left( { - \frac{x - \mu }{\sigma }} \right), if \gamma = 0.} \\ \end{array} , x \ge \mu , \sigma > 0.$$

The extreme value index $\gamma$ is the same as in (8) and the special case $\gamma =0$ represents the exponential distribution. This survival function (10) also represents the exceedance frequency $\Lambda \left(x\right)$ of the aforementioned (approximated) Poisson point process in (6), including for $x < \mu$.

According to respective research (Meerschaert et al. 2012; Albrecher et al. 2021; Raschke 2020), some types of insurance claims can be better represented by the tempered Pareto distribution than by the GPD (10). Therefore, a tempered variant, sometimes also called tapered (Schoenberg and Patel 2012), can be used. The survival function of the tempered GPD is (tempered by an exponential function):

11 $$\overline{H }\left(x\right)={exp\left(-\frac{x-\mu }{\beta }\right)\left(1+\gamma \frac{x-\mu }{\sigma }\right)}^{-1/\gamma },\gamma >0, x\ge \mu , \beta >0, \sigma >0.$$

According to (8) and (11), we formulate a corresponding distribution for the maxima (modified GEV)

12 $$G\left(x\right)=exp\left(-exp\left(-\frac{x-\mu }{\beta }\right){\left(1+\gamma \frac{x-\mu }{\sigma }\right)}^{-1/\gamma }\right).$$

This formulated distribution is not as max-stable via a linear transformation as (8). Nonetheless, the sample of the maxima of (12) also has a CDF that follows (12). However, it is reparametrised with a transformation parameter *p* with:

13 $$p=\frac{\sigma (1-{n}^{\gamma (1-p)})}{\gamma \beta \text{ln}\left(n\right)}.$$

The reparameterisation can be solved iteratively. The subsequent reparameterisation of (12) is

14 $${\gamma }_{n}=\gamma ,{\beta }_{n}=\beta ,{\mu }_{n}=\mu -p\beta \text{ln}\left(n\right)=\mu -\frac{\sigma }{\gamma }\left(1-{n}^{\gamma \left(1-p\right)}\right),{\sigma }_{n}=\sigma {n}^{\gamma \left(1-p\right)}.$$

Its derivation is a simple manipulation of equations and can be validated heuristically. For the modelling by means of conditional distributions (4), we use the logarithmic normal distribution (lognormal), with standard deviation $\sigma$ and expectation $\mu$ of the logarithm of the random variable (PDF):

15 $$f\left(x\right)=\frac{1}{\sqrt{2\pi }\sigma x}exp\left(-\frac{\left(\text{log}(x) -\mu \right)}{2{\sigma }^{2}}\right), x>0.$$

As a further alternative, we consider the Weibull distribution (PDF):

16 $$f\left(x\right)=\frac{\alpha }{\sigma }{\left(\frac{x}{\sigma }\right)}^{\alpha -1}exp\left(-{\left(\frac{x}{\sigma }\right)}^{\alpha }\right), x>0.$$

For complements, we apply the empirical distribution function (CDF):

17 $$\widehat{F}\left({x}_{i}\right)=\frac{i}{n+1}.$$

Other distribution models could also be used, but we decided not to include additional alternatives for the following reasons. We initially considered the GPD (see (10)) as well as gamma distribution during a first research phase, but the fit was very poor according to Akaike’s information criterion (Lindsey 1996) and visual comparisons. We underline that the GPD also approximately represents the tail of many other distributions according to EVT (Coles 2001; Beirlant et al. 2004). Therefore, other distributions for $G(x)$ in (1) are not systematically considered.

Moreover, the GEV distribution (8) and its modification (12) are preferred for $G(x)$ in (1) for practical reasons. They already imply the probability mass and can be easily applied to maxima of periods both shorter or longer than one year, according to parameter transformation (9, 13, 14). This reparameterisation also offers the possibility to adjust a parametrised model to a new period with a higher frequency of cyber incidents and corresponding losses. The $n$-time higher cyber risk is considered directly by $n$ in (9, 13, 14). Additionally, the GEV distribution implies the frequency function $\Lambda \left(x\right)$ of the point process of losses with explicit parametrisation.

We highlight that the approach with separate, conditional CDF $F(x)$ in (4) is not equal to the common models for loss severity since we analyse and model annual maxima, not all losses as they occur and are considered in common severity models. Nevertheless, we could consider more alternatives as we did in the beginning of our research, but they did not result in an improvement. As an example, the well-known Gamma distribution results in extreme parameters that cannot be handled numerically. The parameters of the Gamma distribution can be derived from the moments of the fitted logarithmic normal distribution. There is a further argument against the approach with separate, conditional CDF $F(x)$ in (4). Potential distribution models, such as the logarithmic normal distribution, have a density $f\left({x}_{T}=0\right)=0$. This is not reasonable when there is also a probability mass at ${x}_{T}=0$. Otherwise, loss severity models with two modes should be more popular.

### Statistical inference

The point estimates are computed by a simple numerical optimisation of the likelihood functions (2) or (5). The corresponding standard errors are quantified by the Bootstrap procedure (Davison and Hinkley 1997). We use the PP plots with general distributed margins to evaluate if the model fits visually. Therein, we transform empiric models to uniform distributed margins to provide comparability between the different distribution models.

With a view to goodness-of-fit tests, our survey data pose two challenges. First, the accuracy of the estimated cyber incident losses is limited, since there are more frequently integers and rounded numbers. These generate little clusters in the data, which are not considered in the models. Thus, a quantitative test might reject the model because of the limited precision of the observations, but not because of principal inadequacy of the fitted models. Nevertheless, we apply Chi-Square goodness-of-fit tests (Snedecor and Cochran 1992) because they can, in contrast to alternatives like Anderson–Darling and Kolmogorov–Smirnov goodness-of-fit tests, better deal with the clusters in the data. However, the Chi-Square test also has limitations, because the result of the test depends on the selected bins to which the observations of interest must be assigned. Second, the aphorism ‘all models are wrong’ (e.g. Box 1976) implies that a statistical test should reject the model for very large samples from real-world data. Since our sample size is relatively large, we assume quantitative testing can only have limited expressive power for our analysis.

We sort out less suitable models using Akaike’s information criterion (AIC) (Lindsey 1996) for model selection. We do not use the Bayesian information criterion (BIC), since the number of loss-free observations is high and depends on data interpretation. We would have different debatable variants of BIC values and avoid this issue by the sole application of AIC. This approach is similar to the model selection for the generalised truncated exponential distribution (Raschke 2015). To calculate our distribution models, we use Excel VBA and Stata 16.

## Empirical results

### Description of modelled subsamples

**Fig. 2:**
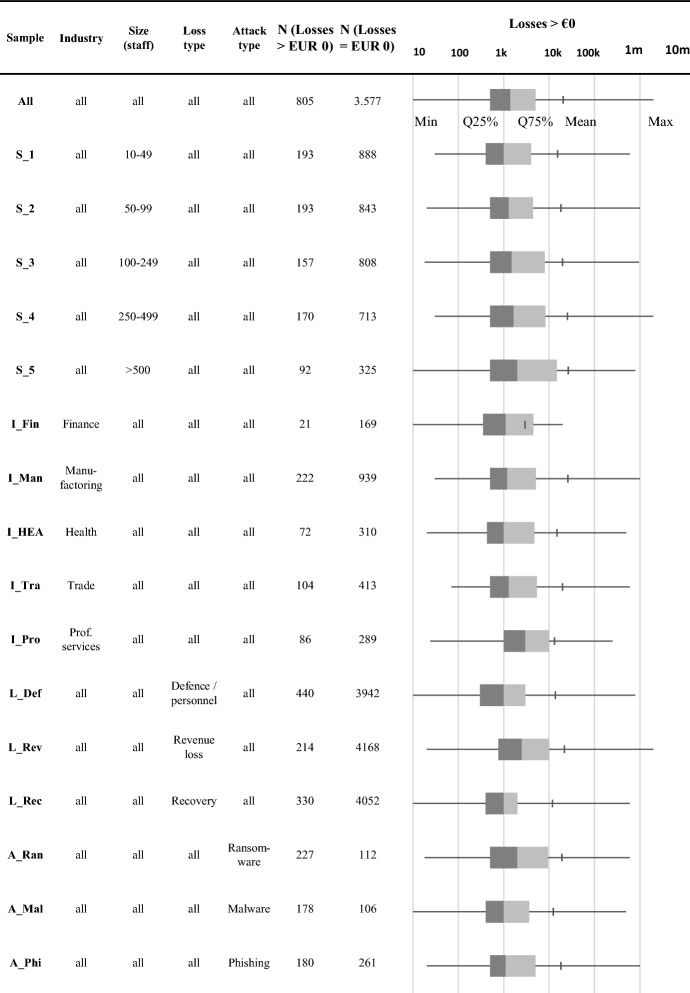
Losses (> EUR 0) of most severe cyber incident in the last 12 months by subsample, box plot and sample description

In order to control for certain company characteristics, we split our sample (All) into 16 subsamples (S_1 to A_Phi) when analysing our data (Fig. 2). Out of 5,000 organisations surveyed, 4,382 organisations gave valid answers (no ‘don’t know’ or ‘not specified’ responses) with regard to experienced cyber incidents. Of those, 3,577 stated no attacks were experienced or no losses incurred. The remaining 805 organisations across all industries and employee classes reported losses between EUR 10 and EUR 2,005,150 (mean: EUR 20,348; median: EUR 1,400; Q.25: EUR 500; Q.75: EUR 5,000). In addition to describing which observations are included in which subsample, we have reported the number of observations that report or do not report losses of cyber incidents. Table 6 in Annex C reports the group comparisons of the mean losses of the different subsamples, using two-sample Wilcoxon rank-sum (Mann–Whitney) tests.

Differences can be recognised between the subsamples. As the number of employees of an organisation increases, so do the median and mean losses (S_1: EUR 1,000 / EUR 15,341; S_2: EUR 1,300 / EUR 18,172; S_3: EUR 1,500 / EUR 19,878; S_4: EUR 1,650 / EUR 25,557; S_5: EUR 2,000 / EUR 26,592), while the mean losses between very large and very small companies are statistically significantly different (see Table 6). However, this effect does not apply to the maximum loss, since the subsamples S_4 (EUR 2,005,150), S_2 (EUR 1,000,000) and S_3 (EUR 960,000) show the highest values and S_5 (EUR 800,000) and S_1 (EUR 600,600) show the lowest maximum loss. With a view to the various industries, financial services in particular stand out with a low mean value and low maximum loss compared to other industries (I_FIN: EUR 2,952 / EUR 20,000; I_Man: EUR 26,051 / EUR 1,000,000; I_HEA: EUR 15,017 / EUR 505,000). However, the mean losses differ statistically significantly only with respect to the health (I_Hea) and professional services sectors (I_Pro; see Table 6).

If we look at the types of losses, it is primarily the losses from revenue shortfalls that are noticeable and statistically significant. Median, mean and maximum losses are almost twice as high, compared to costs for defence/personnel or recovery (L_Rev: EUR 2,500 / EUR 21,829 / EUR 2,005,000; L_Def: EUR 1,000 / EUR 13,818 / EUR 800,000; L_Rec: EUR 1,000 / EUR 11,832 / EUR 600,000). The total losses of the 805 organisations that reported losses sum up to EUR 16,380,099. Of those, defence and investigation/personnel costs make up the largest share (37%), followed by business interruption/revenue losses (29%) and replacement and recovery (24%). Costs for external advice and support (6%), drain of financial means (4%) and compensations and fines (0.5%) represent a smaller proportion of the total losses.

Losses due to ransomware attacks are twice as high compared to malware and phishing attacks, looking at median losses, but are comparable to the mean losses of phishing attacks (A_Ran: EUR 2,000 / EUR 19,269; A_Mal: EUR 1,000 / EUR 12,402; A_Phi: EUR 1,100 / EUR 18,278).

Overall, all subsamples show that there are large ranges of cyber losses, but the median is between EUR 1000 and EUR 3000, indicating most organisations that report a most severe incident suffer smaller losses and few organisations suffer high losses.

### RQ2: Estimated parameters and corresponding fit

We estimated the parameters by the maximum likelihood method and listed their values in Table 2. Focusing on the AIC, the values for the GEV and modified GEV are very similar and, with the exception of the finance subsample (I-Fin), provide the best model variants. However, the model for the overall sample is best estimated using the modified GEV.

**Table 2:** Estimated parameters and corresponding AIC (*lowest value per column)

Model	Parameter	All	S_1	S_2	S_3	S_4	S_5	I_Fin	I_Man	I_HEA
Losses ≤ 0	P	0.8163	0.8215	0.8137	0.8373	0.8075	0.7794	0.8895	0.8088	0.8115
Lognormal	Mue	7.4617	7.1972	7.3673	7.4631	7.6400	7.8831	6.7955	7.5181	7.2870
	Sigma	3.8467	3.6645	3.6236	3.9301	3.5825	4.6409	3.4721	4.1791	3.5867
	*AIC*	*19,569*	*4597*	*4642*	*3867*	*4168*	*2299*	*509*	*5425*	*1721*
Weibull	Alpha	4872.24	3688.03	4302.09	4912.97	5622.74	8051.65	2142.22	5459.29	4000.49
	Sigma	0.4532	0.4529	0.4590	0.4535	0.4595	0.4494	0.6485	0.4296	0.4615
	*AIC*	*19,835*	*4668*	*4710*	*3916*	*4228*	*2320*	508*	*5504*	*1747*
GEV	Mue	− 719.16	− 530.71	− 725.05	− 697.37	− 818.11	− 722.29	− 1134.96	− 537.64	− 558.34
	Sigma	124.86	94.44	145.56	94.95	160.38	131.71	205.84	84.60	103.88
	Gamma	1.3820	1.3519	1.2792	1.4237	1.3650	1.6820	0.7811	1.5804	1.3783
	*AIC*	*19,535*	4577*	4630*	3860*	4160*	*2298*	*509*	*5403*	1716*
Modified GEV	Mue	− 671.92	− 487.62	− 688.61	− 636.63	− 785.70	− 561.55	− 49.89	− 481.70	− 514.09
	Sigma	107.62	79.13	130.86	77.28	147.66	73.46	0.00	66.53	86.69
	Gamma	1.4587	1.4411	1.3339	1.5225	1.4080	2.0322	6.2055	1.7080	1.4756
	Beta	926,391	611,030	1,137,370	743,097	2,117,772	298,439	5269	766,552	506,410
	*AIC*	19,531*	4577*	*4631*	3860*	*4161*	2296*	*509*	5402*	*1717*

		I_Tra	I_Pro	L_Def	L_Rev	L_Rec	A_Ran	A_Mal	A_Phi
Losses ≤ 0	P	0.7988	0.7707	0.8996	0.9512	0.9247	0.3304	0.3732	0.5918
Lognormal	Mue	7.5145	7.9857	6.9597	7.9092	6.9056	7.7443	7.1927	7.3476
	Sigma	3.7623	3.2747	3.4284	3.4474	2.9966	3.7339	3.4965	3.8415
	*AIC*	*2521*	2129*	*10,778*	5973*	*8204*	*4895*	*3670*	*4001*
Weibull	Alpha	5132.87	7211.54	2837.05	7001.99	2548.76	6279.59	3584.23	4332.66
	Sigma	0.4553	0.5713	0.4496	0.5029	0.4630	0.4815	0.4709	0.4528
	*AIC*	*2557*	*2140*	*10,963*	*6026*	*8360*	*4957*	*3732*	*4060*
GEV	Mue	− 476.97	− 1826.9	− 589.36	− 1801.14	− 668.37	0.0000	0.0000	− 354.37
	Sigma	93.62	635.73	49.65	61.42	47.89	974.93	563.98	363.98
	Gamma	1.5412	1.0298	1.2292	1.2026	1.1028	1.3526	1.2984	1.3408
	*AIC*	2506*	*2130*	10,743*	*5974*	8171*	*4894*	3661*	3989*
Modified GEV	Mue	− 413.43	− 1394.8	44.62	− 1716.56	− 653.54	0.0000	− 0.0001	− 337.18
	Sigma	71.21	378.07	− 564.98	53.35	45.10	925.55	545.61	338.89
	Gamma	1.6892	1.3512	1.2705	1.2445	1.1240	1.4635	1.3732	1.4079
	Beta	513,355	130,960	1,118,684	1,910,028	1,648,993	490,909	434,741	882,324
	*AIC*	2506*	*2130*	*10,744*	*5975*	*8173*	4893*	3661*	*3990*

Bold figures are the lowest AIC value per column. Thus, according to the AIC, this model is prefered against others

**Fig. 3:**
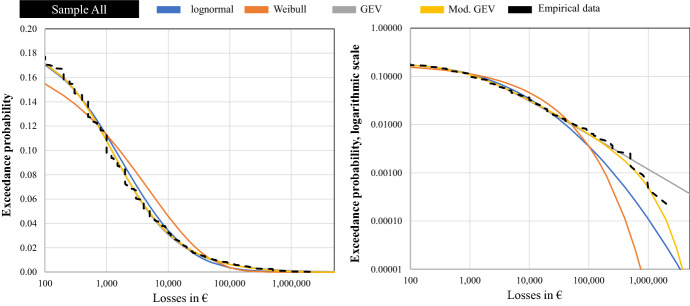
Survival functions of sample: all (y-axis without/with logarithmic scale)

To visually compare the modelled distributions, we plotted the survival functions for the overall sample (Fig. 3). Graphically, the distributions are close together, with the exception of the Weibull distribution. If the same diagram is plotted with a logarithmic scale, it can be seen that the modified GEV in the tail is much closer to the empirical data than the other distributions. Survival functions plots of the subsamples can be found in Annex B.

The fact that we analyse a weighted sample for ‘All’, even though the actual share of organisation classes in the population is different, is not significant. A mixture of the GEV of sample S_1 to S_5 with weighting according to the underlying population (S_1: 79.1%; S_2: 10.5%; S_3: 6.5%; S_4: 2.2%; S_5:1.8%; see Dreissigacker et al. 2020) results in a very similar distribution as our estimate. The relative biggest differences are in the upper tail for losses larger than three million with a 22.5% smaller exceedance probability of the mixture (all: 0.0535%; mixture: 0.0414%).

**Fig. 4:**
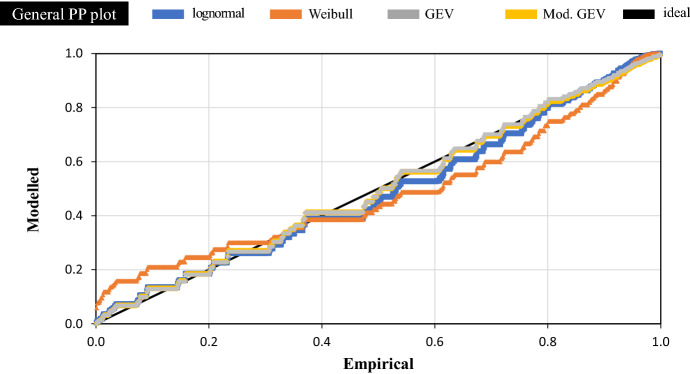
PP plots with general distributed marginals (sample: all)

To validate the fit of the modelled distributions with the empirical data, we plotted probability–probability (PP) graphs with general distributed margins (Fig. 4). The PP plots confirm the scoring of the AIC insofar as the lognormal and Weibull distributions deviate more from the ideal line. The GEV and modified GEV distributions, however, are very similar and follow the ideal line more closely, indicating a better fit. The modified GEV most closely models the rightmost tail of the empirical data. This is particularly important for operational risk management because it is the very seldom but very expensive events that have a lot of influence on the objectives of an organisation.

In addition to the consideration of the AIC as well as visual comparison of the models, we applied Chi-Square goodness-of-fit tests (Snedecor and Cochran 1992) for the overall sample. We distributed 10 classes of observations along the empirical data and shifted them so that any data clusters and class boundaries did not overlap (e.g. EUR 1000 loss observations did not split into two classes). The Chi-Square test statistics also indicate that the GEV and the modified GEV best model the empirical data, with the modified GEV providing a statistically significant fit (Table 3).

**Table 3:** Chi-Square goodness-of-fit test of sample ‘all’; loss classes used: EUR 0, < EUR 1000, < EUR 10,000, < EUR 50,000, < EUR 100,000, < EUR 200,000, < EUR 500,000, < EUR 750,000, < EUR 1,000,000, < EUR 2,000,000; ≥ EUR 2,000,000

Model	Degrees of freedom	Critical value (*α* = 5%)	Chi^2^ statistic
Lognormal	7	14.0671	232.9380
Weibull	7	14.0671	302.2843
GEV	7	14.0671	16.9542
Modified GEV	6	12.5916	11.8191*

*Significant model (*α* = 5%)

### RQ3: Calculation of losses based on the modified GEV

Based on the modified GEV distribution, which implies a loss function and an exceedance frequency (also see the “Data analysis approach, distribution models and inference” section), it is possible to derive losses of single organisations but also of the entire economy. The annual cyber incident loss is the product of the expected loss frequency and the (conditional) expectation of a cyber loss, according to the Wald equation (Wald 1944). For any organisation in Germany with more than nine employees, we expect, based on the modified GEV, an average loss of EUR 3,648 per year. For organisations with 10–49 employees, the loss amounts to EUR 2,445. For the 372,599 organisations of the German economy with more than nine employees, the expected average direct cyber incident loss per year amounts to EUR 1.35 billion (Table 4).

**Table 4:** Expected average direct cyber incident loss per year, based on the modified GEV

Modified GEV: expected average loss	Sample: all (all organisations, > 10 employees)	Sample: S_1 (10–49 employees)
Individual organisation	EUR 3,648	EUR 2,445
German economy	EUR 1,350 million (372,599 organisations)	EUR 720,604,603 (294,726 organisations)

In contrast, if we simply linearly extrapolate the cyber incident losses of our sample of 4,382 valid observations relative to the structure of organisations in the German economy, average direct losses for the most severe cyber incident in the last 12 months sum up to EUR 1.098 million. This very approximate calculation does not consider the issue of interdependence nor the losses of other non-severe attacks. However, regardless of whether we calculate the losses of our dataset using extreme value distributions or simply extrapolate linearly, our results appear to be below those of other publications. We discuss this further in the following section.

## Discussion

Based on a stratified random sample of 5000 German organisations (data collected in 2018/2019), from which 805 organisations reported losses for the most severe cyber incident in the last 12 months, we have modelled four loss distribution types (lognormal, Weibull, GEV and a modified GEV) for 17 different subsamples. According to the AIC and PP plots, the GEV and modified GEV are most suitable to model direct cyber incident losses. However, our modified GEV seems to outperform the GEV when focusing on the tail behaviour, which is also supported by Chi-Square goodness-of-fit tests. In the following, we discuss our findings by using examples and comparing related research, as well as derive implications of our work for practice and academia.

### Example 1: comparing an extrapolation of maximal cyber losses according to GEV and modified GEV

**Fig. 5:**
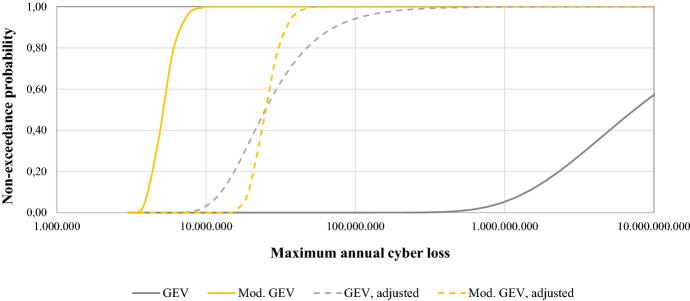
Maximum annual cyber incident loss (PDF) for a single organisation within 370,000 organisations in the German economy (Sample_All)

Even though the modified GEV distributions do not have the best AIC for all subsamples, we are more convinced by these models, since they perform better for the entire sample (Sample_All) and also show significant Chi-Square goodness-of-fit tests. Furthermore, we validate the plausibility of the results by switching the perspective from single organisations to all enterprises. For *n* = 370,000 organisations of the German economy, the distribution of the maximal cyber loss of a single organisation is approximated by ${G\left(x\right)}^{370k}$. We compare the results for GEV and modified GEV in Fig. 5. The median single maximum cyber loss of GEV is EUR 7.5 billion. This seems very high and thus not plausible compared to cyber incident losses of other research (outlined below). The modified GEV distribution has a median of EUR 5.1 million, which seems more reasonable.

Since our results should be validated against other data, we demonstrate how our modified GEV could be easily adjusted, based on further insights on the phenomenon. For example, if we multiply the parameter $\beta$ of the modified GEV distribution (11) by six, we obtain a median of EUR 25 million. We are aware of the issue of interdependence (correlation) between the cyber losses (e.g. caused by untargeted attacks). Such a correlation would be considered by an extremal index *θ* < 1 (Coles 2001, p. 96). However, the interdependence should be relatively small, since the extremal index *θ* is near 1. Otherwise, events with a large number of affected organisations should be much more frequent. Therefore, we neglect this issue in our rough validation.

A modification of the ordinary GEV is also possible by a separate model for its upper tail. We used the Pareto distribution (Beirlant et al. 2004, as a special case of the GPD) for all extremes with exceedance probabilities smaller than 0.002 (loss threshold EUR 485,126). The selected Pareto $\alpha$ is 1.77 (reciprocal of extreme value index $\gamma$). Both illustrative adjustments have a median of around EUR 25 million, which might be a sufficient assumption. However, the adjustment of the GEV needs two additional parameters (threshold and Pareto $\alpha$).

An estimation method with a higher weighting of the tail of the original sample could result in a more realistic median. The Anderson–Darling distance as a special variant of the ML method (Raschke 2020) might be such an alternative.

In the absence of more detailed information, practitioners (e.g. insurers) could adjust the parameter $\beta$ of our modified GEV, i.e. heuristically, to their own loss expectation, in order to allow more accurate calculations to be obtained. Academia and practice should validate our modified GEV approach and its parameterisation against other representative datasets.

### Example 2: insured cyber loss

Modelling cyber incident losses with a (modified) GEV distribution is advantageous, since the derivation of a claim requirement from an actuarial perspective is straightforward. We demonstrate this by an example. An insurer wants to offer a cyber policy for all loss types for organisations with 10–49 employees. Further, a deductible of EUR 1,000 and a coverage amount (limit) of EUR 500,000 is agreed upon. The insurer must calculate the loss/claim requirement/demand. Thus, the insured loss per cyber damage is

18 $$Insured\; loss=\text{Min}(\text{Max}\left(\text{Loss}-\text{Deductible},0\right),\text{Limit}).$$

**Fig. 6:**
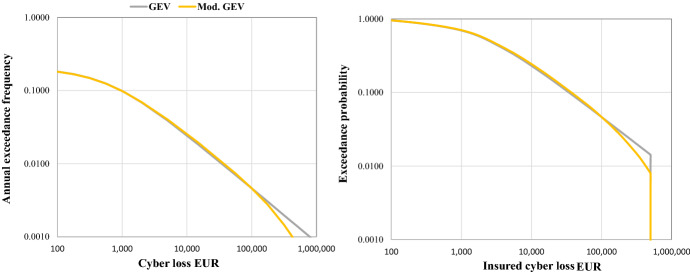
Cyber losses of companies with 10–49 employees: a) exceedance frequency of all loss types, b) distribution of insured cyber losses (deductible: EUR 1,000; limit: EUR 500,000)

The final insurance price also implies the operating costs and the expected profit of the insurer. An additional margin might be also considered. However, as no reliable data on the history of cyber insurance is available so far, the loss/claim requirement/demand must be computed without own experiences. The (modified) GEV distribution implies a frequency function according to (6) as depicted in Fig. 6a. The annual loss/claim requirement/demand is the product of expected claim frequency and the (conditional expectation) of a cyber loss/claim. The expected annual frequency for cyber losses equal to or greater than the deductible of EUR 1,000 is 0.098 for the GEV and modified GEV models. The frequency function is transformed to the (conditional) distribution of insured losses by normalising and shifting, according to the deductible, and cut off at the limit as shown in Fig. 6b. The corresponding (conditional) expectation for the insured loss can be computed numerically, such as by a Monte Carlo simulation. For the example, the expected insured loss is EUR 21,277 (GEV) and EUR 21,306 (modified GEV). Multiplied by the frequency according to the Wald equation (Wald 1944), the annual claim demand per policy is EUR 2,098 (GEV) or EUR 2,100 (modified GEV). The influence of the different tails of the considered maxima distributions is negligible. A further differentiation is conceivable, such as by industry sector or level of cyber protection. The example illustrates how our models can be applied concretely by practitioners and academia.

### The modified GEV in the light of other research

There are further arguments that the modified GEV distribution (12) is an appropriate alternative to model cyber incident losses. It has an exponential tail according to the discussion of Raschke (2020), which implicitly matches with the results of Strupczewski (2019), who analysed large cyber losses worldwide. Strupczewski’s Q-Q plot for the exponential model shows a relative linear pattern for cyber losses larger than USD 100 million, which is characteristic for an exponential tail. Furthermore, he estimated an extreme value index of 0.20264 for the GPD of cyber losses larger than USD 22 million. The corresponding standard error is 0.13122. This implies the difference to the exponential tail with extreme value index 0 is not significant (at the 5% level) for a normal distributed confidence range. In addition, increasing his estimates of Pareto alpha by decreasing order statistics is symptomatic for a tempered/tapered (generalised) Pareto distribution. The example of Albrecher et al. (2021) for tapered Pareto distribution has a similar pattern, which can also be validated heuristically.

### Cyber incident loss estimates reported by other research

In the following, we contrast our loss estimates with the existing literature. A more detailed overview of literature reporting cyber incident losses can be found in von Skarczinski et al. (2022a, b). However, a direct or one-to-one comparison of the losses is not appropriate for two reasons. First, there are few reliable and differentiated data on actual direct cyber incident losses available (Romanosky et al. 2019; Dambra et al. 2020; Eling 2020; Buil-Gil et al. 2021; von Skarczinski et al. 2022a, b). Second, for the literature that exists on the topic, cyber incident losses are systemised and measured differently (i.e. population, sampling, data collection, included losses, time period) because there are no prevailing standards for the operationalisation (Hughes et al. 2017; Paoli et al. 2018). However, to get a sense of our loss estimate, we contrast the results of selected grey and academic literature with ours.

For organisations with more than 1,000 employees, Hiscox (2021) reports a median of USD 24,000 and a 95th percentile of USD 462,000 for the costs of cyberattacks for the last 12 months. The largest cost of a single attack (USD 5.1 million) was reported by a German organisation. However, the operationalisation of the costs as well as the sampling approach of Hiscox is subject to uncertainty. IBM (2020) reports that the global average total cost of a data breach is USD 3.86 million and USD 2.64 million for organisations under 500 employees. German organisations suffer average total data breach costs of USD 4.45 million. Those costs reported include detection and escalation, lost business, notification and ex-post response costs. Here, too, the survey methodology is non-transparent. Bitkom e.V. (2020), a German IT/information association, extrapolates the damages of their survey to the German economy as a whole and arrive at a total economic damage of EUR 205.7 billion for 2018 and 2019. However, with a focus on espionage, sabotage and data theft, they have a somewhat broader focus than our survey. Furthermore, they include additional cost types such as reputational damage, costs for legal disputes, patent infringements and lost business due to counterfeit products and competitive disadvantages. Without these cost types, total losses would be around EUR 82.9 billion for two years (one year ≈ EUR 41.45 billion). Over the course of a year, Bitkom's estimate would thus be around 30 times higher than our extrapolation based on the modified GEV. However, Bitkom records all losses incurred in a year, whereas we only record the most serious cyber incident in a year.

Looking at government and academic research, analysed cyber incident losses appear lower. Richards (2009) surveyed Australian organisations on direct cyber incident costs. Ninety-three per cent of small, 84% of medium and 67% of large organisations suffered costs below AUD 10,000, relating to all incidents of the last year. Median costs are zero, mean costs are AUD 699 and maximum costs are AUD 600,000 for all organisations. Across UK organisations, according to the regular survey of the DCMS (2020), median direct costs (including revenue loss; staff/process/system downtime; lost, damaged or stolen data or assets) relating to the most severe incident in the last year are zero, indicating that most breaches or attacks do not have any material outcomes. Average direct costs for small organisations (median: GBP 0, mean: GBP 580) are lower than for medium/large businesses (median: GBP 0, mean: GBP 1,090). Surveying Belgian organisations, Paoli et al. (2018) found evidence that most organisations did not state profound costs, and only one fifth of affected organisations rated the harm to operational processes as serious or above. With a view to revenue losses, for 60% of the incidents no costs were reported, whilst a further 22.1% stated costs below EUR 10,000.

Although no direct comparison of our loss estimates to the existing literature is possible, implications for practice and academia can be derived. In the light of the fact that grey and academic research differ in the extent of cyber incident losses reported, practitioners should be very careful about which data and modelling base they use for their purposes. There has also been repeated criticism that commercial reports overstate the losses caused by cyber incidents for various reasons (Wolff and Lehr 2017; Paoli et al. 2018; Anderson et al. 2019). Academia, on the other hand, should move towards understanding the factors and causal chains that cause cyber incident losses to occur in organisations (i.e. see von Skarczinski et al. 2021; von Skarczinski et al. 2022a, b), in addition to increased collection of representative real-world data and improved modelling and quantification of loss estimates.

Our research includes limitations that relate largely to the data used. More detailed information on the sample and limitations can be found in Dreissigacker et al. (2020). Given that our study only refers to organisations in Germany, the results cannot necessarily be generalised to organisations in other countries. The sample was drawn on the basis of contact data from two commercial databases and not directly from the population. Although we found no evidence of systematic bias, organisations not included in these databases were thereby also not included in our sample (coverage problem). As with other surveys, the possibility of self-reporting and social desirability bias should be noted. In addition, we retrospectively interviewed only one individual from each organisation, and the interviews were also limited in terms of complexity due to time constraints. The data were collected in 2018/2019. Events that have occurred in the meantime, such as the COVID-19 pandemic, have possibly led to a changed IS situation. However, we assume that even in a pandemic, the fundamental causal relationships of IS have not changed entirely. In addition, no other representative data of comparable scope and detail is available.

## Conclusion

Our article contributes to the body of knowledge by addressing two relevant aspects of cyber risk research.

First, in our opinion, it is the first paper to model cyber losses based on the data of a representative victimisation survey using a stratified random sample. Thus, we offer an alternative to validating the results of prior literature whose analyses are based on op-risk databases that do not allow conclusions about representativeness due to their incompleteness and lack of transparency about the underlying population. In particular, the incidents of smaller organisations, which generate a large part of the European economic output, are not included in op-risk databases, but were included in our survey according to the sampling stratification.

Second, we proposed a modification of the GEV distribution (RQ1), which uses a ‘tempered’ approach to better model the tail of cyber incident losses. Based on our stratified random sample of 5,000 German organisations, from which 805 organisations reported losses for the most severe cyber incident in the last 12 months, we compare our modified GEV distribution to other loss distribution types and find the modified GEV is most suitable to model direct cyber incident losses when focusing on the tail behaviour (RQ2). Based on the modified GEV, we derive expected average losses for any organisation in Germany with more than nine employees of EUR 3,648 per year. For the 372,599 organisations of the German economy that have more than nine employees, the expected average direct cyber incident loss per year amounts to EUR 1.35 billion (RQ3).

We discussed application examples and the comparison of loss estimates with grey and academic literature and derived implications for practice and academia. Our findings could support corporate risk management making IS investment decisions, insurers pricing their policies, or the government as a basis for setting regulatory frame conditions. Although our research has provided novel and significant insight into cyber incident losses, we encourage future work to validate our findings based on further empirical data.

## Figures and Tables

**Fig. 7:**
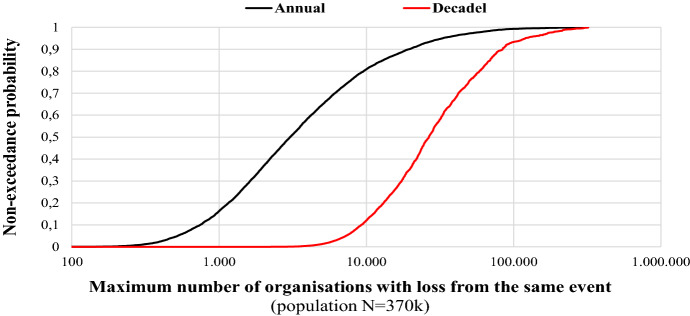
Distribution of maximum number of organisations affected by the same event

**Fig. 8:**
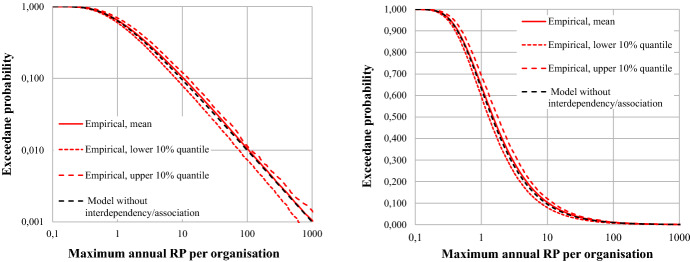
Distribution of maximum return period (RP) of 10,000 companies with interdependence versus exact distribution for the case without interdependency/association (left: logarithmic scale; right: absolute values)

**Fig. 9:**
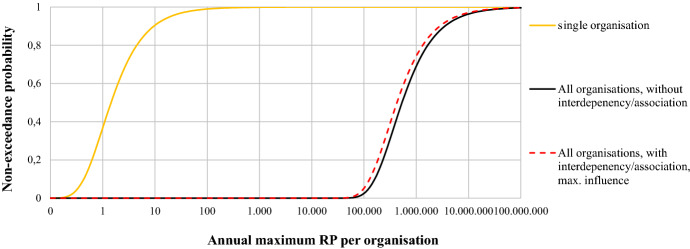
Distribution of maximum RP per company per year for a single company and for the entire population (size 370,000)

## Data Availability

The data used in this article have already been published in other own-project publications. However, the modelling of cyber losses is original and initially published by this article. Due to the sensitive nature of the collected data, the survey consent form with participants states that only aggregated, anonymised data will be published. Therefore, we cannot make the raw data available.

## Annex

### A. Standard errors

See Table 5.

**Table 5:** Standard errors (SE) and sample means (SM) for modelled parameters of (sub)samples (200 bootstrap samples used)

Model	Parameter	All		S_1		S_2		S_3		S_4		S_5	
		SE	SM	SE	SM	SE	SM	SE	SM	SE	SM	SE	SM
Lognormal	Mue	0.2186	3.8460	0.4344	3.6307	0.4198	3.5831	0.4609	3.9046	0.4359	3.5591	0.6721	4.6084
	Sigma	0.0655	7.4635	0.1341	7.1971	0.1305	7.3703	0.1622	7.4631	0.1486	7.6433	0.2388	7.8745
Weibull	Alpha	399.91	4895.75	654.55	3710.33	697.18	4328.71	952.88	4970.12	1057.38	5696.65	2184.98	8217.09
	Sigma	0.0145	0.4540	0.0311	0.4600	0.0350	0.4684	0.0342	0.4612	0.0445	0.4732	0.0339	0.4561
GEV	Mue	66.19	− 725.23	135.34	− 554.48	165.16	− 757.84	193.05	− 733.89	209.15	− 858.95	205.82	− 750.62
	Sigma	20.54	126.81	45.04	105.16	58.46	163.25	48.75	106.43	67.02	175.91	69.06	146.12
	Gamma	0.0759	1.3803	0.1659	1.3382	0.1489	1.2615	0.1709	1.4104	0.1579	1.3520	0.2040	1.6685
Modified GEV	Mue	66.01	− 674.07	137.95	− 507.95	165.60	− 711.63	185.03	− 654.03	197.68	− 783.44	167.02	− 554.52
	Sigma	20.53	108.32	45.39	88.93	58.45	144.52	44.74	83.67	61.71	146.40	50.37	76.36
	Gamma	0.0943	1.4643	0.2085	1.4385	0.1790	1.3337	0.2231	1.5463	0.1893	1.4587	0.3792	2.1253
	Beta	359,354	948,683	459,891	673,694	1.006 m	1.252 m	548,518	760,585	1.544 m	1.894 m	228,237	326,183

		I_Fin		I_Man		I_Hea		I_Tra		I_Pro	
		SE	SM	SE	SM	SE	SM	SE	SM	SE	SM
Lognormal	Mue	1.0571	3.2103	0.4135	4.1146	0.6503	3.5809	0.5335	3.6866	0.4976	3.2426
	Sigma	0.4501	6.7308	0.1302	7.5132	0.2191	7.3036	0.1808	7.5095	0.1811	7.9796
Weibull	Alpha	795.11	2066.32	908.63	5444.61	1233.7	4202.20	1161.2	5149.61	1398.32	7260.57
	Sigma	0.1497	0.7162	0.0253	0.4372	0.0532	0.4743	0.0418	0.4703	0.0517	0.5801
GEV	Mue	1702.77	− 1963.03	102.55	− 559.12	173.79	− 600.19	169.52	− 516.56	621.51	− 1902.2
	Sigma	754.74	643.76	29.80	91.91	57.35	117.86	53.41	109.67	350.99	704.15
	Gamma	0.5889	0.7019	0.1454	1.5587	0.2215	1.3674	0.2085	1.5134	0.1993	1.0241
Modified GEV	Mue	1032.02	− 428.98	98.37	− 495.38	150.29	− 523.00	148.57	− 423.07	557.59	− 1357.3
	Sigma	180.09	38.24	27.33	71.29	44.93	88.26	45.34	76.84	276.05	388.10
	Gamma	9427.76	774.9117	0.1871	1.7052	0.2850	1.5411	0.2946	1.7395	0.5585	1.5264
	Beta	5.824 m	496,323	301,489	737,880	413,153	511,141	285,695	478,260	19081 m	1886 m

		L_Def		L_Rev		L_Rec		A_Ran		A_Mal		A_Phi	
		SE	SM	SE	SM	SE	SM	SE	SM	SE	SM	SE	SM
Log Normal	Mue	0.2737	3.4257	0.3382	3.4665	0.3135	3.0009	0.3516	3.6712	0.4255	3.4761	0.4736	3.8373
	Sigma	0.0949	6.9616	0.1242	7.9052	0.1000	6.9089	0.1341	7.7361	0.1405	7.2012	0.1358	7.3531
Weibull	Alpha	346.79	2860.70	990.02	7050.62	342.80	2576.68	999.63	6240.19	654.96	3652.39	766.45	4405.72
	Sigma	0.0197	0.4515	0.0486	0.5155	0.0264	0.4668	0.0282	0.4894	0.0346	0.4784	0.0315	0.4584
GEV	Mue	75.30	− 594.43	500.67	− 1849.0	87.37	− 675.16	0.00	0.00	26.32	− 7.02	80.81	− 362.14
	Sigma	13.38	51.14	45.86	69.93	15.03	49.78	151.51	995.53	80.14	573.10	90.02	379.14
	Gamma	0.0960	1.2296	0.1383	1.2066	0.1047	1.1043	0.1166	1.3302	0.1344	1.2877	0.1582	1.3356
Modified GEV	Mue	76.71	− 568.59	444.92	− 1491.6	90.76	− 658.68	0.00	0.00	29.66	− 19.84	78.55	− 339.41
	Sigma	13.62	45.95	28.84	39.20	15.59	46.85	144.53	937.72	78.47	540.24	92.21	346.74
	Gamma	0.1122	1.2749	0.3103	1.4511	0.1183	1.1295	0.1521	1.4632	0.1635	1.3824	0.1994	1.4278
	Beta	365,856	1.136 m	1.643 m	1.745 m	41.94 m	5.029 m	212,471	479,990	274,926	438,254	609,815	935,099

### B. Survival functions of subsamples (selection)

Selected survival functions of subsamples (y-axis with/without logarithmic scale).

### C. Test of mean of cyber losses of subsamples

See Table 6.

**Table 6:** Z-values of two-sample Wilcoxon rank-sum (Mann–Whitney) test (**p* < 0.1; ***p* < 0.5; ****p* < 0.01) grouped by subsample

	S_1	S_2	S_3	S_4	S_5	I_Fin	I_Man	I_Hea	I_Tra	I_Pro	L_Def	L_Rev	L_Rec	A_Ran	A_Mal	A_Phi
S_1	–	− 1.096	− 1.354	**− 2.248****	**− 2.556****	–	–	–	–	–	–	–	–	–	–	–
S_2	–	–	− 0.339	− 1.200	**-1.711***	–	–	–	–	–	–	–	–	–	–	–
S_3	–	–	–	− 0.782	− 1.391	–	–	–	–	–	–	–	–	–	–	–
S_4	–	–	–	–	− 0.848	–	–	–	–	–	–	–	–	–	–	–
S_5	–	–	–	–	–	–	–	–	–	–	–	–	–	–	–	–
I_Fin	–	–	–	–	–	–	− 0.932	2.505**	− 0.925	**− 2.316****	–	–	–	–	–	–
I_Man	–	–	–	–	–	–	–	− 0.777	− 0.059	**− 2.557****	–	–	–	–	–	–
I_Hea	–	–	–	–	–	–	–	–	− 0.780	**− 2.753*****	–	–	–	–	–	–
I_Tra	–	–	–	–	–	–	–	–	–	**2.223****	–	–	–	–	–	–
I_Pro	–	–	–	–	–	–	–	–	–	–	–	–	–	–	–	–
L_Def	–	–	–	–	–	–	–	–	–	–	–	**− 6.495*****	0.433	–	–	–
L_Rev	–	–	–	–	–	–	–	–	–	–	–	–	**− 6.739*****	–	–	–
L_Rec	–	–	–	–	–	–	–	–	–	–	–	–	–	–	–	–
A_Ran	–	–	–	–	–	–	–	–	–	–	–	–	–	–	**− 3.095*****	**− 2.099****
A_Mal	–	–	–	–	–	–	–	–	–	–	–	–	–	–	–	− 0.925
A_Phi	–	–	–	–	–	–	–	–	–	–	–	–	–	–	–	–

### D. Analysis and discussion of interdependence

In the following, we discuss the potential influence of the interdependence of cyber hazards on our distribution models. These analysis and modelling are based on the associated Poisson point process. The spectral representation for extremes by Haan (1984) describes and measures such associations between two point processes. The approach is also known as (pseudo) polar coordinates (Coles 2001). Schlathers’ (2002) second theorem also implies the approach and Raschke (2002) applied it to analyse the catastrophic events in the geographical space.

In detail, we assume a boss process which is a Poisson point process ${\Pi }_{B}$ on $(0,\infty )$ with intensity measure $d\Lambda \left(x\right)={x}^{-2}dx$. This means the random number of point events ${X}_{B}>x$ of one realisation of ${\Pi }_{B}$ following a Poisson distribution (7) with intensity (expectation of the number of events ${X}_{B}>x$):

19 $$\Lambda \left(x\right)={\int }_{x}^{\infty }{x}^{-2}dx=1/x.$$

This implies point event $X$ is its own return period (RP) since its reciprocal is the corresponding exceedance frequency. Subprocesses ${\Pi }_{i}$ can be linked to the boss process for each point event via a positive random variable $Y$ (one realisation per point event and company/subprocess) with expectation $E\left[Y\right]=1$

20 $${X}_{i}= {YX}_{B}.$$

The intensity measure and exceedance frequency is the same as for the boss process in (19).

Any occurred/simulated RP can be transformed to the occurred cyber loss of an organisation (or any other real-world point event), via the risk function. The latter describes the relation between event loss and its RP (or exceedance frequency) and is individual for every organisation. Such separate modelling of marginals and dependence structure is used in the common copula approach.

Furthermore, we assume that the share ${\tau }_{u}=20\%$ of the cyber loss events is generated by untargeted attacks (every organisation of the population is attacked) and ${\tau }_{d}=1-{\tau }_{u}=80\%$ is generated by targeted attacks. The reality is more complicated—only a share of the population (such as an industry sector or users of specific IT systems) might be attacked. However, for a rough approximation, this differentiation level is appropriate, since it has as few parameters as possible. For targeted attacks, the occurrence also follows a Poisson process with (19); however, one separate process for every organisation without connections to the other organisations. For the untargeted attacks, we assume the described associated point processes with a random variable $Y$ in (20), which follows a logarithmic normal distribution (15). The standard variation of $log(Y)$ is defined with $\sigma =\text{2,5}$ and expectation with $\mu =-{2.5}^{2}/2$.

According to the cyber loss statistics (Table 2), we assume a general loss threshold for ${\Pi }_{i}$ of ${x}_{L}=5$, which means that, on average, every five years, an organisation suffers a cyber loss. All other simulated RPs are not related to any loss but are fictitious/abstract point events. For directed attacks, the maximum number of losses per attack is one, since only one organisation is attacked. The number of affected organisations of untargeted attacks can be much higher. Its empirical distribution is computed by a Monte Carlo simulation with 9,000 repetitions for all 370,000 organisations of our population. The boss process ${\Pi }_{B}$ is simulated according to (Schlather 2002). Any realisation of a boss process includes an infinite number of point events. In our case, only the 100 largest events ${X}_{B}$ were simulated. Needed random numbers have been simulated by the Mersenne twister generator. To ensure that only the share ${\tau }_{u}=20\%$ of untargeted attacks is considered in the simulation, a simple modification of the loss threshold ${x}_{Lu}= {x}_{L}/{\tau }_{u}=25$ was conducted. The entire loss frequency per organisation (targeted and untargeted attacks) remains 1/5.

The resulting distribution of a maximum number of affected organisations during one event is shown in Fig. 7. The median for the annual distribution is around 3,000. This seems to be very conservative. For a decade, the lower 10% quantile is around 10,000, which is more than 7% of the entire population. There were no such events in the last 20 years that affected nearly so many organisations (with 10 or more employees) in Germany. The report of cyber working party (Frey et al. 2022) includes a broader discussion on this topic. In summary, the assumed model for associated loss events does not seem to underestimate the interdependencies.

Using this model, the consequences of interdependencies can be computed. An issue might be the consequences for the sampling. Does the sample of annual maxima of cyber losses per organisation interviewed in 2019 represent the distribution of the maxima? To answer this question, we, for 10,000 organisations and 100 years, simulated the maximum RP per organisation (from targeted and untargeted attacks). For each year, the sample of the maximum RP per organisation is sorted and for every position the mean and empirical 10% quantile is computed. It can be compared with the distribution of the annual maximum RP for the case of independence. As shown in Fig. 8, the influence of the interdependency is relatively small, so a possible bias can be neglected. The volatility (range between the 10% quantiles) is probably increased by the share of untargeted attack events with association/interdependence.

The last step is about the scaling of the estimated distribution of maximum cyber loss per single organisation to the maximum loss of the entire population. In extreme value statistics, the issue of interdependency of a block maximum is considered by the extremal index ${\theta }_{EI}$, which is larger than 0 and not larger than 1. For the independent, identical distributed random variables with CDF $F\left(x\right)$, the CDF $G\left(x\right)$ of the sample maximum is simply $G\left(x\right)={F\left(x\right)}^{n}$. In case of interdependence (association), it applies (Beirlant et al. 2004).

21 $$G\left(x\right)={F\left(x\right)}^{n{\theta }_{EI}}$$

The interdependencies act like a reduction of the sample size $n$. This means for our issue that the extremal index is ${\theta }_{EI}\le {\tau }_{d}$; it cannot be smaller than the share of the entire sample without any interdependence—the targeted attacks. We estimate the influence of interdependence conservatively when we set ${\theta }_{EI}={\tau }_{d}$ in (21). Again, we demonstrate the effect for the RP to provide generality. The result is shown in Fig. 9. The maximum effect of interdependency in the scaling of the results per organisation to the entire population is measurable. However, it does not strongly influence the dimension of distribution and, in this way, of our estimation per single organisation being scaled to the entire population.
